# Pennsylvania

**Published:** 1897-08

**Authors:** 


### CONTROL WORK.

*Pennsylvania.* The State Live-Stock Sanitary Board has issued the following regulation relating to dogs in the counties of Columbia and Montour:

*Whereas,* There is reason to believe that the disease known as rabies or hydrophobia exists in the vicinity of Catawissa, and the nature of this disease is such that for the present all dogs within certain limits must be suspected of being capable of spreading it; be it

*Resolved,* By the State Live-Stock Sanitary Board, by the authority conferred by the Act of May 21, 1895, that all dogs in the counties of Columbia and Montour are hereby declared to be in a state of quarantine, and must be strictly confined or firmly secured on the premises of their owners, and not allowed to run at large or enter public highways excepting when led or when muzzled with a well-fitting muzzle that will effectually prevent biting.

**After the South there only remains the Pacific coast for us to touch.**

This quarantine shall remain in force for ninety days or until removed by the State Live-Stock Sanitary Board."

Attention is called to the following section of the above-mentioned Act: "Section 5.—That any persons or person violating any of the provisions of this Act or any regulation of the State Live-stock Sanitary Board, or wilfully interfering with officers appointed under this Act, shall be deemed guilty of misdemeanor and shall, upon conviction, be punished by a fine not exceeding one hundred dollars or by imprisonment not exceeding one month, or both, at the discretion of the Court." All owners of dogs in the counties of Columbia and Montour are requested to observe the above regulation, which was made by the State Live-stock Sanitary Board at Harrisburg, June 15, 1897, and are notified that it will be strictly enforced.

A recent outbreak of furious rabies was noted near Mauch Chunk among seven sheep, all of which died. They would fiercely attack anyone approaching, and bite at objects placed near them. They had been bitten by a rabid dog.

**Railroad fares are in your favor all over the land.**

An outbreak of anthrax, causing the death of some twelve cows near Erie, was effectually controlled by the enforcement of the regulations of the State Veterinary Sanitary Board of Pennsylvania.

The Attorney-General of Pennsylvania has just decided that State authorities are empowered to authorize the shooting or destroying of dogs running at large in counties or districts that are proclaimed as the seat of an outbreak of rabies, and where quarantine measures are in force under the direction of the State Veterinary Sanitary Board.

